# Commentary: Morphologically Distinct *Escherichia coli* Bacteriophages Differ in Their Efficacy and Ability to Stimulate Cytokine Release *In Vitro*

**DOI:** 10.3389/fmicb.2016.01029

**Published:** 2016-06-28

**Authors:** Nicolas Dufour, Marine Henry, Jean-Damien Ricard, Laurent Debarbieux

**Affiliations:** ^1^AP-HP, Hôpital Louis Mourier, Service de Réanimation Médico-ChirurgicaleColombes, France; ^2^INSERM, Infection Antimicrobials Modelling Evolution, UMR 1137Paris, France; ^3^Department of Microbiology, Molecular Biology of Gene in Extremophiles, Institut PasteurParis, France; ^4^Université Paris Diderot, Infection Antimicrobials Modelling Evolution, UMR 1137Paris, France

**Keywords:** phage therapy, safety, pro-inflammatory mediators, endotoxins, purification, immune response

In their recent paper, Khan Mirzaei et al. investigated the pro-inflammatory potential of bacteriophages (Khan Mirzaei et al., 2016). They addressed a crucial question linked to the safety of phage therapy, especially when the administration of bacteriophages is anticipated to be performed on highly reactive body compartments (e.g., the bloodstream by intravenous injection or the lung alveolar area by nebulization).

Using peripheral blood mononuclear cells from healthy donors they measured the release of three cytokines (TNF, IL-6, and IL-10) following incubation with four different bacteriophage solutions. As routinely performed, the positive control consisted in lipopolysaccharide extract (LPS) and the negative control in cell culture medium (low endotoxin controlled medium). When using the highest amount of bacteriophages (10^9^ PFU/well), the authors observed that most of the solutions led to a significant increase in the acute pro-inflammatory cytokines (IL-6, TNF-α), compared to the level obtained with the negative control.

Surprisingly, the four bacteriophages solutions elicited a very high release of pro-inflammatory cytokines, with average values ranging from 0.25 to 1-fold the values obtained with the positive LPS control. In particular, bacteriophage SU63 was found to be as potent as LPS to induce an IL-6 secretion with a value as high as 40,000 pg/mL.

Such results raise the question of the quality of the bacteriophage preparations in terms of endotoxin level. Since a universally approved method for the preparation of bacteriophages for human application (or animals, including those used in experimental phage therapy models) is still lacking, we should make our best efforts to fully document the method used.

Here the authors followed a well-known protocol starting from polyethylene glycol precipitation of a bacterial lysate followed by a cesium chloride (CsCl) ultracentrifugation step and most likely a dialysis against a buffer which composition is not specified. No further purification step seems to have been undertaken before these solutions were tested for their pro-inflammatory potential. We believe and subsequently present supporting data that such a protocol is not sufficient to remove endotoxin contamination from CsCl-purified bacteriophage solutions.

In our laboratory, we use a different protocol based on concentration/washing by ultrafiltration and two CsCl ultracentrifugations (a step gradient followed by an isopycnic gradient) (Henry et al., 2013). After the dialysis step, we perform an affinity chromatography dedicated to endotoxin removal (EndoTrap Blue, Hyglos, Germany). This last step, repeated 3 to 5 times, is easily carried out using commercially available columns and can guarantee, in most cases, a very low level of endotoxin, usually below 0.5 EU/mL.

When we measured the endotoxin level present in solutions dialyzed following CsCl ultracentrifugations, we found them to be quite high (see Figure 1 for 4 independent solutions). It is only after the third passage through the endotoxin removal column that low levels were reached. The use of such endotoxin removal methods applied to bacteriophage solutions were, to our knowledge, first reported in 2004 (Boratynski et al., 2004) and are considered as a required step when producing bacteriophage batches for clinical applications (Merabishvili et al., 2009) or immunological studies (Majewska et al., 2015). In the absence of such appropriate procedures, Cooper et al. (2014) have measured a gigantic level of endotoxin (>1,000,000 EU/mL) in a cocktail of bacteriophages prepared for nebulization and obtained from a crude lysate passed through a rudimentary 0.2 μm filter for sterilization.

**Figure 1:**
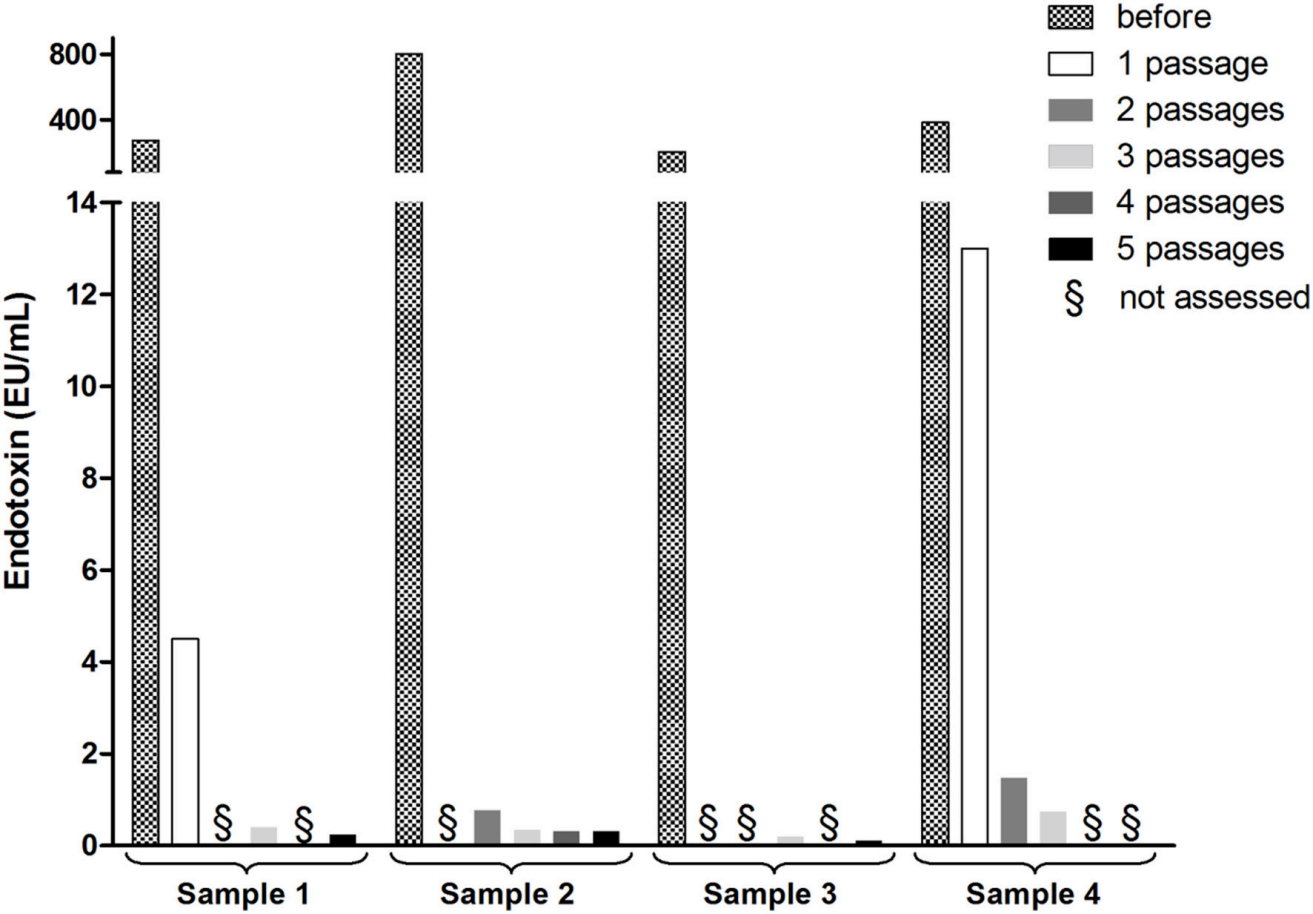
**Endotoxin levels in four independent preparations of bacteriophage PAK-P1 infecting *Pseudomonas aeruginosa* (Henry et al., 2013)**. Limulus amebocyte lysate-based assays (Endozyme rFC assay, Hyglos, Germany) were carried out before and after 1 to 5 passages of the solutions through a specific endotoxin removal column (Endotrap blue, Hyglos, Germany). Before the specific endotoxin removal step, each bacterial lysate (500 mL) was sterilized with two in-line filters (pore sizes, 0.8 to 0.45 and 0.2 to 0.1 μm; Sartopore 300; Sartorius) and concentrated/washed with an ultrafiltration cassette (Vivaflow 200; Sartorius). The concentrates were then ultracentrifuged twice on cesium chloride gradients and dialyzed against Tris buffer (10 mM Tris, 150 mM NaCl, pH 7.5). Final concentration ranged from 10^9^ to 10^11^ pfu/mL. Similar results were observed with coliphages.

Therefore, only purified bacteriophage solutions showing the lowest achievable endotoxin level (which may vary for each bacteriophage preparation), should be used to perform immunological tests. Otherwise, inaccurate conclusions could be made by attributing to bacteriophages an effect that originates from residual endotoxins (or other pro-inflammatory molecules). Moreover, apart from endotoxin, which triggers Toll-like receptor-4 (TLR-4), bacterial lysates may also contain several pathogen-associated molecular patterns (PAMPs) able to elicit a pro-inflammatory response, such as flagellin (sensed by TLR-5), unmethylated CpG Oligodeoxynucleotide DNA (TLR-9), lipoteichoic acid from Gram-positive bacteria (TLR-2) and triacyl lipopeptides (TLR-1 with TLR-2) (Akira and Hemmi, 2003).

We can agree with the concluding remarks from Khan Mirzaei et al., who suggest using ELISA assays to profile the immune response induced by phage formulations in order to provide a sensitive alternative to endotoxin assessment, but only if such a profiling method is formerly bound to an appropriate purification protocol dedicated to potent pro-inflammatory molecules removal. Unfortunately for patients, when bacteria succumb to a viral attack, they do not release a ready-to-use pharmaceutical grade bacteriophage solution!

## Author contributions

ND, JR, and LD wrote this letter, MH performed the experiment provided as example.

### Conflict of interest statement

The authors declare that the research was conducted in the absence of any commercial or financial relationships that could be construed as a potential conflict of interest.
